# Survival of cervix cancer patients in Kampala, Uganda: 1995–1997

**DOI:** 10.1038/sj.bjc.6601034

**Published:** 2003-07-01

**Authors:** H Wabinga, A V Ramanakumar, C Banura, A Luwaga, S Nambooze, D M Parkin

**Affiliations:** 1 Kampala Cancer Registry, Department of Pathology, Makerere University Medical School, Kampala, Uganda; 2 International Agency for Research on Cancer, 69372 Lyon Cedex 08, France; 3 Ministry of Health, Kampala, Uganda; 4 Department of Radiotherapy, Makerere University Medical School, Kampala, Uganda

**Keywords:** cervix, Africa, survival, radiotherapy, treatment

## Abstract

The survival experience of 261 patients with cancer of the cervix registered by the Kampala population-based cancer registry, Uganda, in 1995–1997, is described. Vital status of the subjects was established by active methods including a search of hospital records and house visits. Of the 261 cases, 82 (31.4%) were dead and 105 (40.2%) were alive at the closing date of 31 December 1999; the remaining 74 cases (28.4%) were lost during the follow-up period. Overall observed and relative survival at 3 years was 52.4 and 59.9%, respectively. Of these cases, one-quarter (63) had been treated in the radiotherapy department. These cases had better survival (82.6%) than nontreated patients (78.5%) after 1 year of follow-up, but there was no difference at 3 years. HIV status was not significantly related to prognosis. Stage is an important determinant of survival: cases with distant metastasis had a risk of death some three times that of patients with localised disease. Early detection and prompt treatment should improve overall survival from cervix cancer, in the African context.

Cancer of the cervix uteri is the second most common cancer among women worldwide, with a particularly high incidence in sub-Saharan Africa, where there were an estimated 57 000 cases in 2000, comprising 22% of all cancers in women (Parkin *et al*, 2003). The stage at presentation of tumours in African women is generally very advanced (Schonland and Bradshaw, 1969; Rogo *et al*, 1990; Lomalisa *et al*, 2000) Facilities for the adequate clinical management of those cases that do present at a stage where therapy might be successful are often very inadequate; for example, radiotherapy is available in only 16 of 50 counties in sub-Saharan Africa, and the provision of equipment quite insufficient to deal with the numbers of cancer cases requiring therapy (Levin *et al*, 1997). Nothing is known of the outcome (survival) of women diagnosed with cancer of the cervix in these circumstances. Only a few data from highly selected clinical series have been published (Walker *et al*, 1985), and there has been no attempt to estimate population-based survival for any population on the African continent.

In this paper, we report on a study of survival of women with cancer of the cervix diagnosed among residents of Kyadondo County, Uganda, during the period 1995–1997. Kyadondo County includes the capital city of Uganda, Kampala, as well as surrounding peri-urban and rural areas, with a total surface area of 1914 km^2^ and a population (1997) of 1.2 million. Cancer of the cervix is the most important tumour among women in Uganda, with an age-standardised incidence of 41.7 per 10^5^ in Kyadondo county in 1993–1997 (Parkin *et al*, 2002). We also compare the survival pattern of patients that received radiotherapy treatment and those that did not.

## SUBJECTS AND METHODS

The Kampala Cancer Registry, located in the Department of Pathology at Makerere Medical School, registers all new cancer cases occurring among residents of Kyadondo County. Case finding is by active search for cancers diagnosed in three pathology laboratories, or in six hospitals in the area, or followed up by the Uganda Hospice. Death certificates are rarely completed for deaths occurring outside hospital, and are therefore not used as a source of information. The registry methods and results have been described previously (Wabinga *et al*, 1993,^2000^).

All incident cases of cervix cancer registered during 1993–1997 in the Kampala Cancer Registry were selected for the study of survival. Information abstracted included names, age, residential address, tribe, HIV status, admission date, basis of diagnosis, date of last contact, and status at last contact. HIV status was available for a considerable number of cases in this period because of a separate study of the association between HIV infection and cancer (Newton *et al*, 2001) being conducted in the Kampala hospitals.

Follow-up of these subjects was conducted during 2001–2002 to establish their vital status at the closing date of the study, 31 December 1999. For those cases not known to have died, the first stage in the follow-up required tracing the patients' clinical records in the local hospitals, to retrieve available information on patient status, to complete any that had been missing from the registry record, and to abstract any recorded information on stage of disease at diagnosis (as TNM or FIGO stage). The case file was also linked with the computerised database of the Uganda Hospice using the RECLINK software, to identify those cases that had been referred there for management. (RECLINK is a record linkage software developed at unit of Descriptive Epidemiology, International Agency for Research on Cancer, Lyon. The software performs probabilistic linkage between records from different sources using selected personal identifiers (names, date of birth, sex, address, tribe). The hospice provides outreach palliative care (including counselling, basic chemotherapy, or pain relief) for cancer patients, and follows up all referred cases to them, including those that did not receive any treatment after the initial consultation. The cases for which information on vital status at the closing date was not available from these sources were followed up by home visits. The process is difficult, since formal addresses do not exist in Uganda. The place of residence is recorded simply as the name of the locality; street names and house numbers are not used. In the more rural areas, the local chiefs or leaders were asked for information on the exact place where the patient lived. The offices of lowest level of local government (Local Council II) maintain a list of people living in the area, based on the register of voters. Registration is needed in order to vote, but is not compulsory. If these procedures failed to locate an individual, the project staff made enquiries of other local residents whether they knew about the person in question. In the urban areas, local councillors were usually asked, but since many urban area residents are tenants of rented property, not homeowners, this method was less successful than in rural areas.

Details of all cervix cancer cases referred to the Department of Radiotherapy in the period 1993–1997 were recorded in a computer file, which included patient details, stage of disease, as well as information on the type of therapy given and follow-up. This radiotherapy file was then matched with the file of registered cervix cancer cases from Kyadondo County using RECLINK. In fact, since the Department of Radiotherapy was closed during 1993 and 1994, the final analysis of results of the study comprised only those cases diagnosed during the 3-year period, 1995–1997.

Data entry used the Epi-info™ software and the files were exported to STATA™ for analysis. Cumulative observed probabilities were calculated using the Kaplan–Meier method. To calculate observed survival, death from any cause was taken as failure, and the subjects who were lost to follow-up prior to the closing date, and those known to be alive on the closing date were censored on those dates. Observed survival was also calculated using two other assumptions: that all cases lost to follow-up had died on the date of lost follow-up (minimum survival), or that such patients remained alive until the closing date of the study (maximum survival). Cumulative relative survival probabilities were calculated using Hakulinen's method (Hakulinen and Abeywickrama, 1985). The probability of death, by age, was taken from the Uganda life tables for the year 2000 published by the World Health Organization (WHO, 2000). The log-rank test was used in a univariate analysis to identify potentially important prognostic variables. The variables that showed statistical significance on univariate analysis were introduced stepwise into a Cox regression model to identify the independent predictors of survival. Age standardisation of the relative survival was carried out by the direct method, using six age groups (<35, 35–44, 45–54, 55–64, 65–74, 75+) and the standard patient population provided by Sankaranarayanan *et al* (1998).

## RESULTS

A total of 261 cases of cervix cancer were recorded among Kyadondo residents for the 3-year period 1995–1997. Of these cases, 63 (24%) had been referred to the Department of Radiotherapy. Table 1

**Table 1:** Distribution of cervix cancer cases in Kampala, Uganda, 1995–1997

	**Radiotherapy (63)**	**No radiotherapy (198)**	**All cases (261)**
	**No. (%)**	**No. (%)**	**No. (%)**
Age			
<35 (years)	17 (26.9)	46 (23.2)	63 (24.1)
35–44	15 (23.8)	64 (32.3)	79 (30.3)
45–54	20 (31.8)	42 (21.2)	62 (23.8)
55–64	5 (7.9)	22 (11.1)	27 (10.3)
65+	6 (9.6)	24 (12.2)	30 (11.5)
			
Mean age	44.0	45.5	45.1
			
Stage			
Stage I	17 (27.4)	21 (10.6)	38 (27.9)
Stage II	35 (56.4)	22 (10.6)	57 (41.9)
Stage III	9 (14.5)	19 (12.6)	28 (20.6)
Stage IV	1 (1.6)	12 (6.6)	13 (9.6)
Missing	1 (1.6)	124 (62.6)	125 (47.9)
			
Basis of diagnosis			
Nonhistological	14 (22.2)	79 (39.9)	92 (35.2)
Histology	49 (77.8)	119 (60.1)	169 (64.8)
			
Tribe			
Ganda	41 (65.1)	121 (61.1)	162 (62.1)
Others	22 (34.9)	76 (38.4)	98 (37.5)
Missing	0	1 (0.5)	1 (0.4)
			
Vital status			
Dead	17 (27.0)	65 (32.8)	82 (31.4)
Alive	34 (54.0)	71 (35.9)	105 (40.2)
Lost FU	12 (19.0)	62 (31.3)	74 (28.4)
			
HIV infection			
Yes	21 (33.3)	48 (24.2)	69 (26.4)
No	30 (47.6)	64 (32.3)	94 (36.0)
Missing	12 (19.0)	86 (43.4)	98 (37.6)

shows the distribution of cases by age, stage, basis of diagnosis, tribe, HIV status, and vital status on the closing date of the study. Of the 261 eligible cases, 82 (31.4%) were known to have died, 105 (40.2%) were known to have been alive on the closing date, and 74 (28.3%) had been lost to follow-up. The cases lost to follow-up did not differ from those successfully traced in terms of age (mean ages were 45.6 in those lost and 44.1 in those traced, *P*<0.23), tribe, stage, or HIV status. Only 63 cases (24.1%) had been treated by radiotherapy. The mean age of these cases was 44.0, compared with 45.5 for those not receiving treatment by radiotherapy (*t*=1.81 and *P*<0.04). Stage was clearly important in determining treatment; only 24% of cases in stages III and IV had received radiotherapy, compared with 61% of cases in stage II (who might be expected to profit the most), and 45% in stage I (who would be treated by surgery as the method of choice). The diagnosis in 78% of the radiotherapy cases had been confirmed histologically, while only 60% had been histologically confirmed in the nontreatment group. HIV status was known for 163 (62.4%) of cervix cancer patients; 69 (42.3% of those with known status) were HIV positive. HIV status did not seem to have determined the use, or otherwise, of radiotherapy, although the percentage of cases in which HIV status was known was much higher in the radiotherapy cases (81%) than in those not referred for treatment (56.5%).

With respect to the radiotherapy treatment, only one in four patients (16 out of 63) had received both external beam and intracavity radiation. Almost half of the patients (30 out of 63) received treatment by external beam alone, and nine cases by intracavity radiation alone.

**Figure 1:**
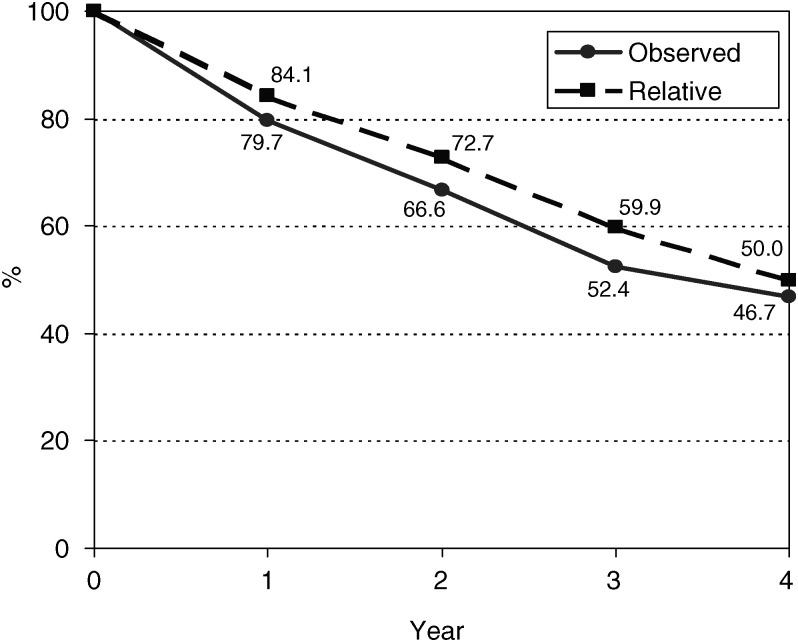
Observed and relative survival from cervix cancer in Kampala, Uganda, 1995–1997

Observed and relative survival is shown in Figure 1

. Overall observed and relative survival at 3 years was 52.4 and 59.9%, respectively. Cases treated by radiotherapy had a significantly better survival in the first year of follow-up (82%) compared with nontreated patients (78.5%), but this advantage had disappeared 2 years post diagnosis. Information on clinical staging was available for 52.1% of cases. Survival declined steadily with advancing stage of disease at diagnosis. Among the cases for which HIV status was known, there was no difference in survival at 1 (*P*=0.09), 3 (*P*=0.23), or 4 years (*P*=0.72) post diagnosis.

Independent predictors of survival using the Cox proportionate hazards model are presented in Table 2

**Table 2:** Independent predictors of death in 261 cases of cancer of cervix uteri, Kampala, Uganda, 1995–1997

Odds ratios and 95% confidence intervals		
**Predictor**	**Univariate**	**Multivariate**
Age (years)		
<35	1.0	1.0
35–44	1.5 (0.9–2.4)	0.9 (0.4–1.9)
45–54	1.6 (1.0–2.6)	1.0 (0.5–2.2)
55–64	2.0 (1.1–3.6)	2.3 (0.8–6.3)
65+	2.2 (1.2–4.0)	1.7 (0.6–5.3)
		
Stage		
Stage I	1.0	1.0
Stage II	0.8 (0.4–1.7)	0.9 (0.4–2.0)
Stage III	1.5 (0.6–3.4)	1.8 (0.7–4.4)
Stage IV	3.1 (1.3–7.5)	3.1 (1.1–8.8)
Unknown	0.4 (0.2–0.8)	0.3 (0.1–0.8)
		
Treatment		
Radio therapy	1.0	1.0
Not treated	0.9 (0.5–1.6)	1.0 (0.5–2.1)
		
Tribe		
Ganda	1.0	1.0
Other	1.2 (0.8–1.9)	1.3 (0.7–2.3)
		
HIV infection		
Positive	1.0	1.0
Negative	0.9 (0.6–1.5)	0.7 (0.4–1.2)

. Stage is the most important determinant of survival: cases with distant metastasis (stage IV) had a risk of death some three times that of patients with localised disease. The somewhat increased risk of death with advancing age seen in the univariate analysis is not present when the other variables (especially stage) are included in the model. Tribe, HIV status, and treatment by radiotherapy were not related to prognosis.

**Figure 2:**
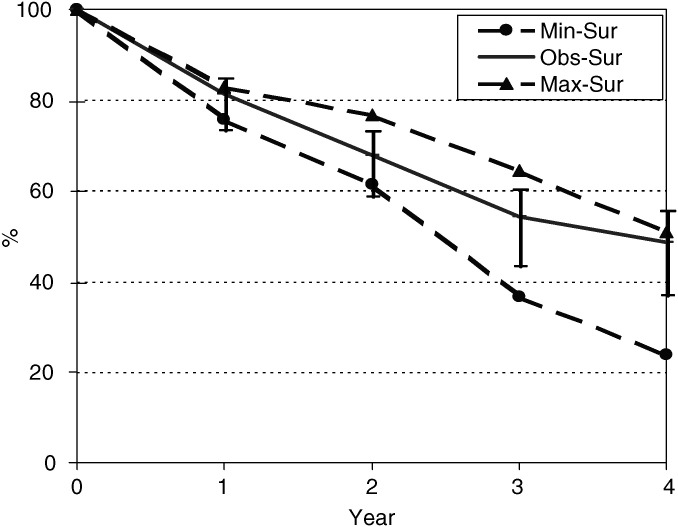
Observed survival (with 95% CI) and estimated maximum and minimum values. Cervix cancer cases, Kampala, Uganda 1995–1997

The possible effects of the loss to follow-up of 74 (28.4%) of cases were examined by calculating a maximum survival (assuming that all were alive at the closing date) and a minimum survival (assuming that all such patients had died at the date of follow-up). Figure 2

shows the observed survival, calculated by the actuarial (life table) method, and minimum and maximum estimates. At 3 years, the observed survival was 54.2%, with a minimum estimate of 36.6% and a maximum of 64.4%.

## DISCUSSION

The patients represented all of the cases diagnosed in the population of Kyadondo County, Uganda, in 1995–1997. The Kampala Cancer Registry was founded in 1954, in the Department of Pathology of Makerere University Medical School. The aim was to obtain information on cancer occurrence in the population of Kyadondo County in which the capital city of Kampala is situated. The registry functioned continuously until 1971, when, because of the unstable political situation, full population coverage became impossible. The registry was restarted (in 1989) and has functioned continuously since. When it began, the registry relied upon request/result forms of the Department of Pathology, which had been redesigned specifically to permit registration of cancers. In addition to data collected in this way, tumour registrars have been employed to search for cancer cases admitted to, or treated in, all of the hospitals in Kampala (and, in recent years, the Uganda Hospice) and, for individuals resident in Kyadondo County, to extract somewhat more extensive information onto special notification forms. The registry file has been maintained on computer since 1989.

Incidence rates, for the population of Kyadondo County, have been published for 1989–1991 (Wabinga *et al*, 1993) and 1993–1997 (in Parkin *et al*, 2002). A complete review of the data set, for four time periods (1960–1966, 1967–1971, 1991–1994, 1995–1997), has been published by Wabinga *et al* (2000). The incidence rate of cervix cancer in women is high, and the rates have increased markedly between the 1950s and 1960s, and the 1990s. This increase is unlikely to be related to the epidemic of AIDS. It is possible that the social disruption of the Amin dictatorship and the following civil wars (1972–1986) favoured the spread of HPV, like other sexually transmitted diseases; HPV was found to be present in the great majority of cervix cancers in Uganda, with HPV type 16 in 53% (Bosch *et al*, 1995).

The results show that survival at 3 years (52.4%) is fair, and certainly not as poor as is often assumed for cervix cancer in Africa (Sherris *et al*, 2002). The result may be compared with 1 and 3 years age-standardised relative survival (0–74 years) in nine other populations (Table 3

**Table 3:** One-year and 3-year age-standardised relative survival (ASRS) from cancer of the cervix in selected populations of women aged 0–74 years

**Country/population**	**Period**	**ASRS (1 year)**	**ASRS (3 years)**
Uganda, Kampala	1995–1997	81.4	49.0
Zimbabwe, Harare^1^	1995–1997	66.1	44.9
			
India, Mumbai^1^	1992–1994	77.4	57.5
China, Qidong^1^	1987–1991	60.1	45.0
Thailand, Khon Kaen^1^	1993–1997	58.5	41.3
			
UK, Northern Ireland^2^	1993–1996	83.0	70.0
Europe^3^	1985–1989	92.1	85.0
			
United States, SEER registries^4^	1995	89.7^5^	78.1^5^
Canada, Ontario^6^	1971–1996	89.3	78.9
			
Australia, New South Wales^7^	1980–1995	89.9	70.9

1 Unpublished results from the registries (see Acknowledgements).

2 N Ireland Cancer Registry (2001).

3 Berrino *et al* (1999).

4 Ries *et al* (2001).

5 Relative survival in SEER population: age-specific data unavailable.

6 Marrett *et al* (1999).

7 NSW Cancer Council (2002).

).

These are the first population-based survival data from Africa. Cancer registration that involves identification of all new cases arising in a defined population is a difficult proposition in Africa (Parkin *et al*, 2003), but the follow-up of all registered cases for periods of several years adds a further degree of complexity. In Kampala, tracing patients at home proved to be quite difficult, since addresses consist simply of a local area; street names and numbers do not exist. In addition, most urban residents retain a connection with their ‘home’ village, to which they may well return, to be with relatives at the time of death. Tracing more than 70% of patients was therefore a reasonable achievement by the project staff. Furthermore, the patients who were not traced did not seem to be a biased subgroup, at least in terms of demographic characteristics, or HIV status. They may, however, have been more (or less) likely to die than the patients who were successfully traced. The maximum and minimum survival estimates provide a range for the true value.

As in other studies from Africa, the stage of disease at diagnosis is relatively advanced, although the percentage of cases in stage 1 (27.9%) is higher than in previously reported series from Africa; it was only 7% among cases treated by radiotherapy in Nairobi, Kenya, in 1974–1979 (Rogo *et al*, 1990) and 8.4% in cases admitted to Johannesburg Hospital in 1997–1998 (Lomalisa *et al*, 2000). The higher percentage of cases in stage I probably reflects both the general level of health awareness in the population and easy access to medical facilities in Kyadondo county. As has been noted in many previous studies (Sankaranarayanan *et al*, 1998), stage is an important determinant of survival from cervix cancer, and the only significant influence on outcome in the present study. The rather low observed survival may also relate to competing causes of death in this population. Uganda was one of the first countries affected by the epidemic of HIV/AIDS, although by the time of this study (1995–1997) prevalence of infection had more than halved since the maximum in the early 1990s, the peak age group for diagnosis of AIDS in women in Uganda is 25–29, and two-thirds of AIDS cases occur under the age of 35 years (Ministry of Health, 2002), while this age group had a lower case fatality from cervix cancer than older women (Table 2). HIV status was known for almost two-thirds of cases, and, although HIV-positive subjects had a rather higher risk of dying than those who were negative, the difference was not significant.

It is perhaps surprising that less than a quarter of the cases had received any radiotherapy; the stage of disease was clearly important in selecting the cases most likely to benefit. HIV positivity is not considered to be a contraindication. The outcome in cases treated by radiotherapy was no better than for those who were not (except possibly in the first year of follow-up).
